# A 20 Bp Indel of *HNF4A* Is Associated with Piglet Growth Partially by Regulating Its Transcription

**DOI:** 10.3390/ani16121797

**Published:** 2026-06-10

**Authors:** Jingtong Huang, Yu Zhang, Yingkun Zhang, Ruhai Xu, Xiaoyu Chen, Xiaohong Chu, Nana Yang, Buyue Niu, Lihe Dai

**Affiliations:** 1 Zhejiang Key Laboratory of Livestock and Poultry Biotech Breeding, Institute of Animal Husbandry and Veterinary Science, Zhejiang Academy of Agricultural Sciences, Hangzhou 310021, China; 17710235879@163.com (J.H.); xurh@zaas.ac.cn (R.X.); chenxy@zaas.ac.cn (X.C.); zhuxh@zaas.ac.cn (X.C.); yangnana0815@163.com (N.Y.); 2 College of Animal Science and Technology, Northeast Agricultural University, Harbin 150030, China; yuzhang1275@163.com (Y.Z.); zyk15137844374@163.com (Y.Z.)

**Keywords:** *HNF4A*, pig, growth trait, molecular marker

## Abstract

Hepatocyte nuclear factor 4α (*HNF4A*) is a critical transcription factor that regulates the differentiation and metabolism of intestinal epithelial cells. However, its role in piglet growth remains unclear. In this study, the tissue expression of *HNF4A* was examined using RT-qPCR, and the putative functional SNPs were analyzed by integrating bioinformatics and DNA sequencing. Association analysis was performed in 156 Min pigs and 160 Landraces, and the biological function of the identified genetic variant was explored using a dual-luciferase reporter assay. The results showed that *HNF4A* was widely expressed in liver, kidney and gastrointestinal tissues, with significantly higher expression in the liver of adult pigs than in newborn piglets (*p* < 0.05). A 20 bp InDel was identified in the first intron of porcine *HNF4A*. Allele frequency analysis showed that the Del allele (20 bp deletion) was dominant in Landrace and Duroc pigs, while the In allele (20 bp insertion) was dominant in Min and Jinhua pigs. Association analysis revealed that Min pigs with the In/Del genotype had significantly higher body weights at 14, 21, 28 and 35 days and higher average daily gain (ADG) than those of the In/In animals (*p* < 0.05). Landrace piglets with the Del/Del genotype exhibited significantly higher body weight at 21 and 28 days than those of the In/Del genotype (*p* < 0.05). The dual-luciferase reporter assay suggested that the plasmid carrying the In allele exhibited higher transcriptional activity than the Del allele (*p* < 0.05). Notably, the genotype associated with superior growth performance differed between the two breeds. Collectively, a 20 bp InDel within *HNF4A* was identified, which might affect piglet growth partially by modulating its transcription, and further study in other populations with different genetic backgrounds is needed before its application in pig breeding.

## Simple Summary

Hepatocyte nuclear factor 4α (*HNF4A*) is a transcription factor involved in intestinal cell differentiation, metabolism and nutrient absorption. In this study, a 20 bp insertion/deletion (InDel) was identified in the first intron of the porcine *HNF4A*, which was associated with piglet body weight and daily gain in Min pigs and Landraces. Furthermore, this InDel altered the transcriptional activity of porcine *HNF4A*, suggesting it might be a potential functional marker in animal selection breeding.

## 1. Introduction

Growth traits are among the most important economic traits, and they are controlled by multiple genes with complex genetic architecture [1,2]. Identification of causal genes or variants has been the foundation for the genetic improvement of growth traits. In the past decade, numerous genetic variants associated with growth traits have been reported using GWAS [3,4,5]. However, most of them have not been functionally characterized, which limits the understanding of the molecular basis of the growth trait.

Nowadays, it is well accepted that functional variants are mainly located in non-coding regions rather than coding regions [6]. These variants regulate gene expression and play roles in shaping complex traits [7]. For example, a 13 bp Indel regulates the expression of the ribosomal protein S27-like (*RPS27L*) by modifying the binding of transcription factor 3 (TCF3) and myogenic differentiation antigen (MYOD), thereby promoting myoblast proliferation and ultimately affecting pig body weight and backfat thickness [8]. SNP rs702045770 (g.539G>A) within porcine SYNPO2 intron sense-overlapping lncRNA (*pSYISL*) regulates the binding of Yin Yang 1 (YY1) to the promoter, thereby leading to the differential expression of *pSYISL* and variation in backfat thickness or muscle fiber density in different populations [9]. A 14 bp Indel in the promoter of porcine mannose receptor C type 1 (*MRC1*) is associated with different responses to porcine circovirus type 2 (PCV2) infection, partially by regulating the transcription of *MRC1* [10].

Hepatocyte nuclear factor 4α (*HNF4A*) is a nuclear receptor transcription factor that plays a crucial role in regulating metabolism [11], differentiation, and proliferation of intestinal epithelial cells [12,13]. It is highly abundant in the liver, where it governs gluconeogenesis and lipid metabolism [14]. Its dysregulation is linked to metabolic diseases such as type 2 diabetes [15] and non-alcoholic fatty liver disease (NAFLD) [16]. Beyond its well-documented functions in the liver, an increasing number of studies have verified its essential roles in intestinal tissues. Intestinal epithelium serves as the primary site for intestinal lipid absorption and metabolism, and *HNF4A* precisely modulates fatty acid uptake, intracellular lipid transport, and apolipoprotein synthesis within enterocytes. In murine models, *HNF4A* mediates fatty acid oxidation in intestinal stem cells, and its deletion impairs stem cell renewal and epithelial regeneration, thereby reducing nutrient absorption and body weight gain [17]. Moreover, *HNF4A* directly activates a panel of brush border genes involved in carbohydrate and lipid transport across the intestinal epithelium, a process critical for postnatal growth [18]. In humans, GWAS have linked *HNF4A* variants not only to inflammatory bowel diseases but also to altered fasting glucose levels and lipid profiles, suggesting its pleiotropic role in energy balance and growth [19,20]. Despite these mechanistic insights in rodents and humans, the specific genetic variations within the porcine *HNF4A* and their relationship with piglet growth remain largely unexplored. Recently, Xiang et al. [21] identified porcine *HNF4A* as one of the identified transcription factors enriched within differential open chromatin regions (OCRs) in the duodenum of Large White pigs with divergent feed efficiency, using integrated RNA sequencing (RNA-seq) and ATAC sequencing (ATAC-seq). These differential OCRs were associated with genes involved in glycolytic and fatty acid processes, which are tightly linked to energy metabolism and growth performance in pigs. Collectively, these findings suggested that *HNF4A* might be a candidate gene for porcine growth traits. Nevertheless, functional characterization of genetic variations within the porcine *HNF4A*, as well as their direct roles in growth regulation, is still limited. Addressing this knowledge gap is essential for the potential application of *HNF4A* variants as molecular markers in pig breeding programs.

In this study, the tissue expression of *HNF4A* was analyzed; then, the functional genetic variations in porcine *HNF4A* were screened by integrating in silico and DNA sequencing and the association analysis between specific variations and growth traits were performed in Min pigs (a Chinese indigenous breed) and the Landrace; lastly, the biological function of the putative functional genetic variation was verified using a dual-luciferase reporter assay, which might contribute to the genetic improvement of piglet growth traits.

## 2. Materials and Methods

### 2.1. Animals

For the tissue expression analysis, samples including liver, spleen, stomach, kidney, duodenum, jejunum, ileum, colon, and cecum were collected from four one-month-old pigs as described by Niu et al. [22]. Nine Min pigs raised in the Lanxi Farm (Lanxi, Heilongjiang, China) were slaughtered at three developmental stages (birth, one-month-old, nine-month-old), and the tissues of liver, duodenum, jejunum, and ileum were collected. For the association analysis, all the Min pigs (*n* = 156) and Landraces (*n* = 160) were born and raised on Lanxi Farm (Lanxi, Heilongjiang, China) under identical feeding and management conditions, ensuring consistent environmental effects, and the two breeds were raised concurrently during the same period. These animals were weaned at day 35 and weighed at birth, day 3, 7, 14, 21, and 35, then the average daily gain (ADG) was calculated as follows: ADG = (35 days’ weight-birth weight)/35. Additionally, Jinhua pigs (*n* = 87) were raised in the Zhejiang Mebolo Swine Breeding Farm (Jinhua, Zhejiang, China), and Durocs (*n* = 299) were provided by Haining Yangdu Science and Technology Ranch of Zhejiang Academy of Agricultural Sciences (Hangzhou, Zhejiang, China).

### 2.2. In Silico Analysis

SNPs of porcine *HNF4A* were retrieved from the dbSNP database of Ensembl (https://www.ensembl.org/index.html, accessed on 10 December 2025), and putative functional non-synonymous SNPs (ns-SNPs) were predicted using the Polymorphism Phenotyping v2 tool (http://genetics.bwh.harvard.edu/pph2/, accessed on 10 December 2025), SNAP software 13.0.0 (https://github.com/KorfLab/SNAP, accessed on 10 December 2025), SIFT (http://sift-dna.org), and PhD-SNP (https://snps.biofold.org/phd-snp/phd-snp.html, accessed on 10 December 2025). A phenome-wide association study (PheWAS) for *HNF4A* was performed using the PigBiobank database (https://pigbiobank.farmgtex.org/, accessed on 10 December 2025) [23]. Cell-type localization and gene expression patterns of porcine *HNF4A* were analyzed using the Integrated Pig Gut Cell Atlas (IPGCA) database, which was accessed via the scGutDB online visualization platform (http://alphaindex.zju.edu.cn/scgut, accessed on 10 December 2025) [24]. Partial genomic sequences of porcine *HNF4A* from different breeds were retrieved from the Ensembl database (https://www.ensembl.org/index.html, accessed on 10 December 2025), and these sequences were aligned using DNAMAN 9 software (https://www.lynnon.com/) and Chromas 2.2.0 (https://technelysium.com.au/wp/chromas/, accessed on 10 December 2025).

### 2.3. Quantitative Real-Time PCR (RT-qPCR)

Total RNA was extracted from tissues using TRIzol, reverse-transcribed into cDNA through PrimeScript RT Master Mix (Takara, Dalian, China). According to the manufacturer’s instructions of the TB Green^®^ Premix Ex Taq™ II (Takara, Dalian, China), the RT-qPCR reaction (20 μL) consisted of 100 ng of cDNA, 10 μL of SYBR mixture, and 0.2 μM of primers HNF4A-D or GAPDH-D (Table 1). The reaction was carried out on the ABI QuantStudio 3 system (Applied Biosystems, Foster City, CA, USA) with 95 °C/30 s; 40 cycles of 95 °C/5 s and 60 °C/35 s; and melting curve analysis (60–95 °C, 0.3 °C/s). The RT-qPCR was performed in technical triplicate. Relative mRNA expression of *HNF4A* in liver, spleen, stomach, kidney, duodenum, jejunum, ileum, colon, and cecum was calculated with the 2^−ΔΔCt^ method against glyceraldehyde-3-phosphate dehydrogenase (GAPDH) as an internal reference gene.

### 2.4. Genotyping of Genetic Variants in Porcine HNF4A

The primer pairs used in this study were designed using Primer 5 software based on the DNA sequences of porcine *HNF4A* (ENSSSCT00000008067.5). Each reaction volume (10 μL) for PCR consisted of DNA templates (100–150 ng), 5 μL of 2 × Taq Master Mix (Takara, Dalian, China), and 0.5 μM of primers HNF4A-C or HNF4A-1 (Table 1). The amplification conditions consisted of a denaturation at 94 °C for 4 min, followed by 35 cycles of denaturation at 94 °C for 30 s, annealing at 55 °C for 30 s, extension at 72 °C for 45 s, and a final extension at 72 °C for 5 min. The PCR products containing this SNP were amplified by primers HNF4A-C-F/R, purified, and sequenced commercially (Sangon, Shanghai, China).

To genotype the 20 bp InDel of *HNF4A*, HNF4A-2F/R was designed (Table 1). The PCR reaction was performed in a total volume of 20 μL, which included 100–150 ng of DNA, 10 μL of 2 × Taq Master Mix (Takara, Dalian, China), and 0.5 μM of primers. The PCR reaction conditions were as follows: pre-denaturation at 94 °C for 5 min, followed by 35 cycles of denaturation at 94 °C for 30 s, annealing at 55 °C for 30 s, and extension at 72 °C for 35 s, and finally an extension at 72 °C for 5 min.

### 2.5. Plasmid Construction

Using the genomic DNA of Del/Del or In/In pigs as templates, the promoter of *HNF4A* was amplified by primer HNF4A-3F/R (Table 1). The PCR reaction was described above, and the products were separated, purified, and sequenced commercially. Then, the verified product was digested with *Hin*dIII, cloned into pGL3-Basic vector (Promega, Madison, WI, USA) using the homologous recombination enzyme 2 × CE Mix (Vazyme, Nanjing, China). After DNA sequencing and double digestion verification, the correct plasmids were identified and designated pGL3-HNF4A-Del/Del and pGL3-HNF4A-In/In.

### 2.6. Cell Transfection and Luciferase Reporter Gene Assay

Small intestinal epithelial cells (IPEC-J2) were cultured in a medium composed of high-glucose DMEM (Gibco, Grand Island, NY, USA), 10% fetal bovine serum (Gibco, Carlsbad, CA, USA), and 1% penicillin-streptomycin (Beyotime, Shanghai, China), incubated at 37 °C and 5% CO_2_, and seeded into 24-well plates. When the cell density reached approximately 60%, 1.5 μL of LP2000 transfection reagent (Invitrogen, Carlsbad, CA, USA) was used to transfect 0.5 μg of pGL3-HNF4A-In/In, pGL3-HNF4A-Del/Del, or pGL3-basic, together with 0.005 μg of pRL-TK into the cells, respectively. After 24–36 h, all the cells were collected and lysed according to the instructions of the Dual-Luciferase Reporter Assay Kit (Beyotime, Shanghai, China). The activities of firefly luciferase and Renilla luciferase were measured using a Sirius L Luminometer (Berthold, Pforzheim, Germany), and the ratio of firefly luciferase activity to Renilla luciferase activity (Fluc/Rluc) was calculated. The transfection experiment was performed in triplicate and repeated three times. In each independent experiment, all transfections were carried out in triplicate technical wells. Data are presented as mean ± standard error of the mean (SEM) from the three biological replicates.

### 2.7. Statistical Analysis

Statistical analyses were performed using IBM SPSS Statistics 26 (IBM, Armonk, NY, USA). One-way analysis of variance (ANOVA) was used to analyze the mRNA expression of *HNF4A* in different tissues. A two-tailed Student *t*-test was used to assess differences in gene expression or transcriptional activity between groups. All data were presented as mean ± standard error. Association analysis was conducted using the General Linear Model (GLM) in SAS version 9.4 (SAS, Cary, NC, USA), and the model was as follows:

*Y_ij_* = *μ* + *G_i_* + *S_j_* + *e_ij_*,

where *Y_ij_* is the observed trait, *μ* is the mean of the trait, *G_i_* is the genotype effect, *S_j_* is the maternal effect of dam ID, and *e_ij_* is the random residual. The threshold *p*-value for significance was adjusted after correction for multiple comparisons using the Bonferroni correction (*α*_altered_ = *p*/*n*, where *p* = 0.05, *n* = the number of InDel markers analyzed in this study). Given that only one functional InDel marker was tested in the present study, the corrected significance threshold was set at *α*_altered_, and statistical significance was set at *p* < 0.05, and *p* < 0.01 was considered highly significant. Each experiment was independently repeated at least three times.

## 3. Results

### 3.1. mRNA Expression of Porcine HNF4A in Different Tissues

**Figure 1:**
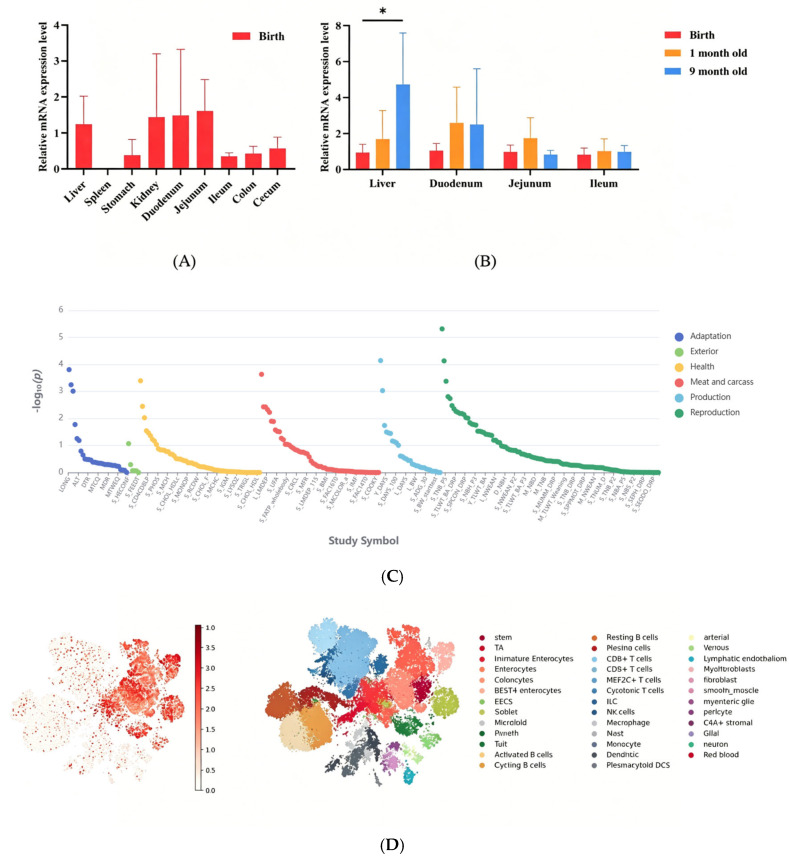
The mRNA expression of porcine *HNF4A* in different tissues (*n* = 3–4). (**A**) Expression of porcine *HNF4A* in gastrointestinal tissues. (**B**) Tissue expression profile of porcine *HNF4A* at different developmental stages. (**C**) The results of *HNF4A* PheWAS. (**D**) Cell-type-specific expression of *HNF4A* in porcine intestine. * *p* < 0.05.

RT-qPCR analysis revealed that *HNF4A* mRNA was ubiquitously expressed in the liver, stomach, kidney, duodenum, jejunum, ileum, colon, and cecum of newborn piglets (Figure 1A). At birth, the highest expression levels were observed in the small intestine, particularly the duodenum, jejunum, and ileum. To further investigate the developmental expression dynamics of *HNF4A*, we examined its transcript levels in the liver and intestinal tissues across three key stages: birth, 1 month, and 9 months of age (Figure 1B). Notably, hepatic *HNF4A* expression increased significantly with age, peaking at 9 months and showing a significant difference compared with birth (*p* < 0.05). In the duodenum, jejunum, and ileum, *HNF4A* expression also exhibited an upward trend during postnatal development. Collectively, these findings demonstrate that *HNF4A* is predominantly expressed in the liver and small intestine, the primary organs responsible for nutrient metabolism and absorption in pigs. PheWAS analysis using PigBiobank [23] linked *HNF4A* to porcine growth traits, including body weight, average ADG, and daily weight gain (Figure 1C, Table 2), which suggests that *HNF4A* may contribute to piglet growth by functioning in small intestinal epithelial cells, affecting nutrient absorption, metabolism, or intestinal health. Additionally, single-cell analysis through IPGCA/scGutDB [24] showed that *HNF4A* was primarily located in small intestinal epithelial cells, including small intestinal stem cells, enterocytes, goblet cells, BEST4 enterocytes, Paneth cells, and tuft cells (Figure 1D).

### 3.2. In Silico Analysis of Ns-SNPs in Porcine HNF4A

**Figure 2:**
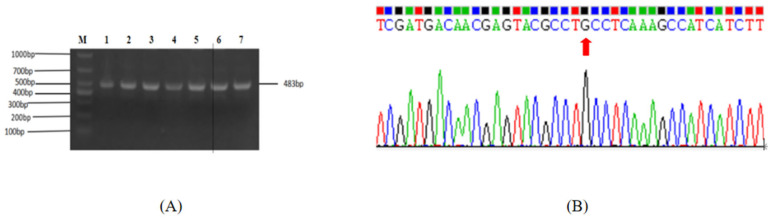
Characterization of SNP: rs3470460054. (**A**) Amplification of the partial porcine HNF4A coding region. (**B**) Sanger sequencing chromatogram of the SNP: rs3470460054.

By retrieving the dbSNP Database, a total of 29 SNPs were found in the coding region, including six ns-SNPs. Among them, only rs3470460054 (C288Y) was predicted to be deleterious by SNAP, SIFT, and PhD-SNP (Table 3). The 483 bp fragment including SNP: rs3470460054 was obtained randomly from five Min pigs and five Landraces (Figure 2A); however, this SNP did not exist in these animals (Figure 2B). The results indicated the functional SNPs might be located in the transcriptional regulatory region instead of the coding region.

### 3.3. A 20 Bp InDel Was Identified in Porcine HNF4A

**Figure 3:**
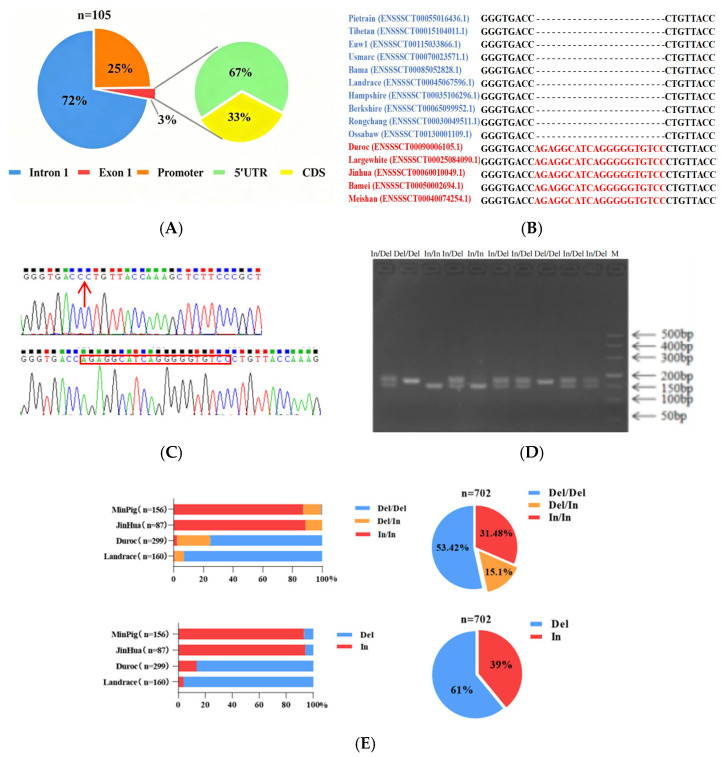
Identification and analysis of the 20 bp InDel in Porcine *HNF4A*. (**A**) Proportion Chart of SNPs in the Porcine *HNF4A* Promoter, first exon, and First Intron Sequences. (**B**) Identification of a 20 bp InDel in the Intron of porcine *HNF4A*. (**C**) Sequencing results of the 20 bp InDel in the intronic region of the porcine *HNF4A*. The box indicates the 20 bp inserted fragment, and the arrow points to the InDel site. (**D**) Genotyping results of the 20 bp InDel. Lane M: Trans DNA Marker I. (**E**) Genotype and allele frequencies of InDel among different pig breeds.

A partial genomic sequence of *HNF4A* (including the first exon, first intron, and the 2000 bp 5′ flanking sequence) from 15 breeds was retrieved from the Ensembl database. And 105 single-nucleotide polymorphisms (SNPs) were found in this sequence. Among them, the majority (72%) were located in the first intron, followed by the promoter region (25%), while only 3% were found in the first exon. Further partitioning of the non-intronic variants revealed that 67% were located in the 5′UTR and 33% in CDS. Compared with Western breeds such as Landrace, Hampshire, Berkshire, and Pietrain, most of the local breeds like Jinhua, Meishan, and Bamei pigs had a 20 bp insertion (AGAGGCATCAGGGGGTGTCC) in the first intron of *HNF4A* (Chr17: 46,821,378–46,821,397 bp) (Figure 3B). The 2386 bp fragment of the *HNF4A* promoter of Min pigs and Landraces was amplified using primers HNF4A-1F/R. Sequence alignment revealed the existence of the predicted InDel (Figure 3C). The PCR products amplified by primers HNF4A-2F/R showed three band patterns after agarose gel electrophoresis: a single 152 bp band representing the Del/Del homozygote (deletion allele, resulting from the absence of the 20 bp sequence), a single 172 bp band representing the In/In homozygote (insertion allele, containing the 20 bp sequence), and both bands (152 bp and 172 bp) representing the In/Del heterozygote (Figure 3D).

Three genotypes were observed in Landrace, Duroc, and Min pig populations. In the Landrace population, the frequencies of Del/Del, In/Del, and In/In genotypes were 0.931, 0.063, and 0.006, respectively, and the frequencies of Del and In alleles were 0.962 and 0.038. In the Duroc population, the genotype frequencies were 0.753, 0.224, and 0.023, respectively, and the allele frequencies were 0.865 and 0.135. In the Min pig population, the genotype frequencies were 0.006, 0.122, and 0.872, respectively, and the allele frequencies were 0.067 and 0.933. In Jinhua pigs, only In/Del and In/In were observed with frequencies of 0.115 and 0.885, respectively, and the frequencies of Del and In alleles were 0.057 and 0.943 (Table 4). The Del/Del, In/Del, and In/In genotypes accounted for 53.42%, 15.1%, and 31.48% of all individuals, and the In and Del alleles accounted for 61% and 39% (Figure 3E). In Landrace and Duroc, the Del allele was predominant, whereas in Min and Jinhua pigs, the In allele was predominant.

### 3.4. The 20 Bp InDel Was Associated with Piglet Growth Trait

Association analysis showed that Min pigs with the In/Del genotype had higher body weight than In/In genotype at 14 days old, 21 days old, 28 days old, and 35 days old (*p* < 0.05). The ADG of In/Del genotype was significantly higher than that of In/In individuals (*p* < 0.01) (Table 5). In the Landrace population, the body weights of In/In individuals at 21 days old and 28 days old were significantly higher than those of In/Del genotype individuals (*p* < 0.05). Additionally, the body weight of Del/Del genotype individuals at 35 days old (*p* = 0.12) and the daily weight gain showed an increasing trend compared with those of In/Del genotype individuals (*p* = 0.09) (Table 6).

### 3.5. The 20 Bp Indel Affects Porcine HNF4A Transcriptional Activity

**Figure 4:**
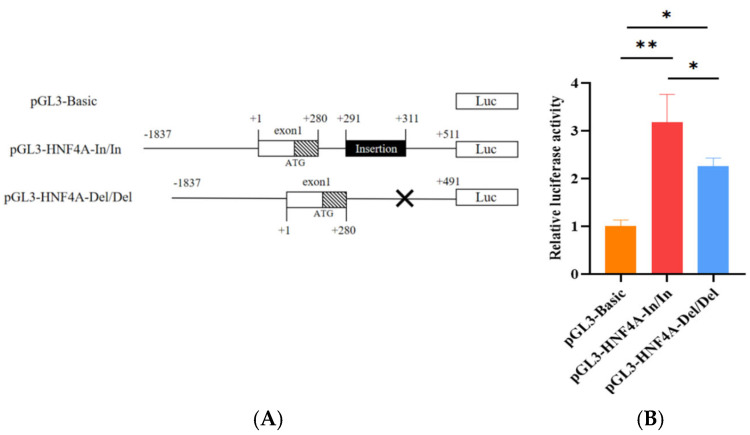
Transcriptional activity and differential expression of the porcine *HNF4A* with 20 bp InDel. (**A**) Schematic diagram of the plasmid construction for a 20 bp InDel in the intronic region of the porcine *HNF4A*. (**B**) Relative luciferase activity of the porcine *HNF4A* 20 bp InDel in IPEC-J2 cells. Data are mean ± SE. * *p* < 0.05, and ** *p* < 0.01. X indicates deletion.

Using the DNA of Del/Del or In/In animals as templates, two fragments (2348 bp and 2328 bp) were amplified with primers HNF4A-3F/R and inserted into to construct pGL3-HNF4A-In/In and pGL3-HNF4A-Del/Del, respectively (Figure 4A). A dual-luciferase reporter assay revealed both the pGL3-HNF4A-Del/Del and pGL3-HNF4A-In/In plasmids exhibited luciferase activity, which was significantly higher than that of the negative control (*p* < 0.05). The luciferase activity of pGL3-HNF4A-In/In was higher than that of pGL3-HNF4A-Del/Del (*p* < 0.05) (Figure 4B).

## 4. Discussion

In this study, *HNF4A* was found to be widely expressed in the liver, kidney, and gastrointestinal tract of piglets. Analysis of external independent databases, including PigBiobank [23] and scGutDB [24], revealed that the porcine *HNF4A* exhibited abundant expression in intestinal epithelial cells, especially enriched in enterocytes, suggesting that *HNF4A* might affect piglet growth by regulating nutrient absorption. Studies in mice have shown that *HNF4A* drives the expression of brush border genes involved in nutrient absorption of the intestine [18]. Given the essential role of the small intestine in nutrient uptake and the observed cell-specific expression pattern, *HNF4A* was selected as a candidate gene for swine growth traits, which might influence piglet weight by regulating intestinal nutrient absorption and metabolic processes.

Genetic variations within the coding region are widely recognized to alter protein structure or function, resulting in phenotype variation [25,26]. For example, rs81403974 and rs325492834 in long non-coding RNA muscle growth-promoting factor (*lncMGPF)* influenced porcine backfat thickness and loin muscle area by altering RNA stability and secondary structure [27]; the g.153G>A mutation in porcine deleted in azoospermia-like (*DAZL*) affected sow fertility by modifying the RNA-binding domain of protein [28]; the c.1226A>G mutation in porcine dual oxidase 2 (*DUOX2*) induces thyroid deficiency in pigs by altering the binding site of serine/arginine-rich (*SR*) proteins [29]. In the present study, rs3470460054 was predicted to be deleterious by SIFT, SNAP, and PhD-SNP; however, this variant was not detected in the animals used in this study. This result may be due to (1) the limited sample size used in the present study, which weakens the detection efficiency of rare variants; (2) the relatively narrow genetic background of the examined pig populations. Therefore, its potential functional role remains to be validated in broader populations. Accordingly, the present study focused on the non-coding regions of porcine *HNF4A* to identify potential regulatory variants that might contribute to animal growth.

By the dbSNP database and DNA sequencing, a novel 20 bp InDel in the first intron of porcine *HNF4A* was identified. Population genetic analysis revealed a breed-specific allelic distribution: the Del allele (deletion) was dominant in Landrace and Duroc pigs, whereas the In allele (insertion) was more prevalent in Min and Jinhua pigs. Interestingly, no Del/Del homozygote was detected in Jinhua pigs (*n* = 87), and only one Del/Del individual (0.6%) was found in Min pigs (*n* = 156). The extremely low frequency of the Del/Del genotype in these breeds may reflect population-specific evolutionary or breeding histories, and genetic drift in relatively small populations might have contributed to the reduced frequency of this genotype [30]. Association analysis indicated that Min pig In/Del individuals exhibited higher pre-weaning body weight and average daily gain than In/In piglets (*p* < 0.05 and *p* < 0.01). In Landrace pigs, the Del/Del genotype was significantly correlated with higher weaning body weight (*p* < 0.05). Two explanations may account for this heterogeneity. Firstly, this phenomenon could be attributed to the distinct selection histories of the populations. Western commercial breeds have been intensively selected for growth rate and feed efficiency, whereas Chinese indigenous breeds have undergone survival selection for adaptability and robustness. Secondly, distinct linkage disequilibrium (LD) structures underlie the breed-specific associations. For example, European pig breeds exhibit long-range LD (up to 400 kb) and large haplotype blocks, while Chinese indigenous breeds show rapid LD decay (generally ≤ 10 kb) [31]. Thus, the Del allele (deletion) is favorably linked to growth-promoting variants in Landrace, while in Min pigs, the In allele (insertion) or the In/Del heterozygote captures the advantageous combination due to a different LD phase and faster LD decay. Based on these breed-specific patterns, we propose distinct breeding strategies: in Min pigs, introducing the Del allele may improve early growth; in Landrace, maintaining or increasing the Del allele frequency is beneficial; and in Duroc, gradual reduction in the In allele may enhance population uniformity. Nevertheless, further validation in larger populations is required before applying this marker in breeding programs.

Nowadays, it is widely accepted that non-coding regions harbor abundant functional variations that regulate gene expression [32,33]. In the present study, a 20 bp InDel located in the first intron of porcine *HNF4A* significantly affected transcriptional activity. Specifically, the insertion allele (In) exhibited higher activity than the deletion allele (Del) in the luciferase assay, suggesting a regulatory role at the transcriptional level. This finding is consistent with previous studies showing that intronic variations can modulate transcriptional activity by altering local regulatory elements. For example, Ren et al. [34] identified a 12 bp InDel in the porcine *Oct4* (Octamer-binding transcription factor 4), in which homozygous males exhibited superior reproductive traits compared with heterozygotes. However, association analysis in Min pigs revealed the opposite: In/Del piglets grew significantly faster than In/In piglets. This seemingly contradictory observation can be resolved by the non-linear relationship between regulatory activity and phenotypic outcome, which has been well recognized [35]. One possible explanation is that, in In/In homozygotes, two highly active In alleles may drive *HNF4A* protein levels excessively high. Such an overexpression may trigger cellular feedback inhibition or accelerate protein degradation, thereby reducing net functional output. In contrast, In/Del heterozygotes carry only one In allele, and their *HNF4A* expression level likely falls within an optimal range for function. This pattern is consistent with the general principle of non-linear gene dosage effects [36], although the specific molecular mechanisms require further investigation. At the pre-transcriptional level, the higher activity of the insertion allele suggests that this InDel might influence transcriptional activity by altering the transcription factor binding, DNA methylation, chromatin accessibility, or their interaction. However, post-transcriptional regulation of *HNF4A* remains unknown. Future studies incorporating larger and more diverse populations, together with integrative analyses combining gene expression profiling and in vivo functional validation, will be necessary to further elucidate the biological role of this variant.

## 5. Conclusions

Porcine *HNF4A* is expressed in intestinal tissues; the 20 bp InDel in the first intron of this gene partially affected piglets’ weight by regulating its transcription. This mutation might be a molecular marker for selection breeding, which contributes to improving the efficiency of the swine industry. However, further validation in larger and diverse independent pig populations is essential to verify its genetic stability before practical breeding application.

## Figures and Tables

**Table 1:** Primers used for RT-qPCR, SNPs identification, and plasmids construction.

Primer Name	Primer Sequence (5′→3′)	Annealing Temperature	Product Size (Bp)
HNF4A-D	F: CTACATCATCCCTCGGCACT	60 °C	199
	R: CTGGGAACGCAGCCTCTT		
GAPDH-D	F: CCCCAACGTGTCGGTTGT	60 °C	83
	R: CCTGCTTCACCACCTTCTTGA		
HNF4A-C	F: ATCTGTAAAATGGGTGTG	52 °C	483
	R: AGCCTCTGGAGTAAGTGC		
HNF4A-1	F: CATCCGAATAGTGATAGAGGTA	55 °C	2328
	R: TCCCAAAGACACCCTGAAA		
HNF4A-2	F: GAATGACAGACGAGCCCG	55 °C	172
	R: CAGGACGCCAAGAGGAAG		
HNF4A-3	F: TAAGTAAGCTTCATCCGAATAGTGATAGAGGTA	55 °C	2328
	R: ATGCCAAGCTTTCCCAAAGACACCCTGAAA		

The underline shows the additional restriction sites: *Hin*dIII (AAGCTT).

**Table 2:** Significantly associated traits in the HNF4A PheWAS production category results.

Gene	Trait	Study Symbol	*p*-Value	Category
*HNF4A*	Body weight (end test)	Y_BW	7.2040 × 10^−5^	Growth
*HNF4A*	Days	Y_DAYS	3.1885 × 10^−2^	Growth
*HNF4A*	Average daily gain	Y_ADG	9.3235 × 10^−4^	Growth

**Table 3:** Prediction of functional ns-SNPs of porcine HNF4A.

SNP ID	Substitution	Polyphen-2		SNAP		SIFT		PhD-SNP	
		Prediction	Score	Prediction	Score	Prediction	Score	Prediction	Score
rs3472612586	N262S	Neutral	0.87	Neutral	0.77	Neutral	1	Neutral	0.72
rs3473595056	V266M	Neutral	0.75	Neutral	0.77	Neutral	0.2	Neutral	0.66
rs3472730409	V281I	Neutral	0.87	Neutral	0.83	Neutral	1	Neutral	0.78
rs3470460054	C288Y	Neutral	0.64	Deleterious	0.56	Deleterious	0.02	Deleterious	0.86
rs346061759	R432Q	Neutral	0.64	Neutral	0.50	Neutral	0.6	Neutral	0.89
Srs691705449	A462S	Neutral	0.61	Neutral	0.58	Neutral	0.3	Neutral	0.83

**Table 4:** Genotype and allele frequency of the 20 bp InDel in porcine *HNF4A*.

Breed	Number	Genotype Frequency			Allele Frequency	
		Del/Del	In/Del	In/In	Del	In
Landrace	160	0.931 (149)	0.063 (10)	0.006 (1)	0.962	0.038
Min pig	156	0.006 (1)	0.122 (19)	0.872 (136)	0.067	0.933
Jinhua	87	0	0.115 (10)	0.885 (77)	0.057	0.943
Duroc	299	0.753 (225)	0.224 (67)	0.023 (7)	0.865	0.135

**Table 5:** Association analysis between the 20 bp InDel of *HNF4A* and the growth trait in Min pigs.

Traits	Genotype			*p*-Value
	Del/Del	In/Del	In/In	
Number of individuals	1	19	136	
Birth weight/kg	-	1.04 ± 0.05	1.06 ± 0.02	0.608
Weight at 3 days of age/kg	-	1.28 ± 0.06	1.26 ± 0.02	0.720
Weight at 7 days of age/kg	-	1.75 ± 0.09	1.75 ± 0.03	0.977
Weight at 14 days of age/kg	-	2.69 ± 0.14	2.33 ± 0.05	0.020
Weight at 21 days of age/kg	-	3.86 ± 0.21	3.34 ± 0.08	0.021
Weight at 28 days of age/kg	-	5.02 ± 0.26	4.35 ± 0.10	0.016
Weight at 35 days of age/kg	-	6.31 ± 0.32	5.51 ± 0.12	0.019
Average daily weight gain/(kg/d)	-	0.15 ± 0.01	0.13 ± 0.00	0.009

Bonferroni correction was applied (α = 0.05/*n*, *n* = number of InDel markers = 1).

**Table 6:** Association analysis between the 20 bp InDel of *HNF4A* and the growth trait in Landrace.

Traits	Genotype			*p*-Value
	Del/Del	In/Del	In/In	
Number of individuals	149	10	1	
Birth weight/kg	1.49 ± 0.03	1.46 ± 0.10	-	0.790
Weight at 3 days of age/kg	1.80 ± 0.03	1.84 ± 0.11	-	0.759
Weight at 7 days of age/kg	2.44 ± 0.04	2.33 ± 0.16	-	0.507
Weight at 14 days of age/kg	3.83 ± 0.08	3.35 ± 0.31	-	0.125
Weight at 21 days of age/kg	5.29 ± 0.12	4.33 ± 0.44	-	0.037
Weight at 28 days of age/kg	6.89 ± 0.15	5.57 ± 0.60	-	0.035
Weight at 35 days of age/kg	8.43 ± 0.19	7.17 ± 0.79	-	0.120
Average daily weight gain/(kg/d)	0.20 ± 0.01	0.16 ± 0.02	-	0.091

Bonferroni correction was applied (α = 0.05/*n*, *n* = number of InDel markers = 1).

## Data Availability

The dataset used and analyzed during the current study is available from the corresponding author upon reasonable request.
